# A cross-sectional study to identify a set of risk factors for caprine herpesvirus 1 infection

**DOI:** 10.1186/s12917-018-1401-8

**Published:** 2018-03-14

**Authors:** S. Bertolini, A. Rosamilia, C. Caruso, C. Maurella, F. Ingravalle, A. Quasso, P. L. Acutis, M. Pitti, L. Masoero, G. Ru

**Affiliations:** 1 Istituto Zooprofilattico Sperimentale del Piemonte, Liguria e Valle d’Aosta, Turin, Italy; 2 Istituto Zooprofilattico Sperimentale dell’Abruzzo e del Molise G. Caporale, Teramo, Italy; 3 Local Health Unit AT, Asti, Italy; 4 Local Health Unit TO4, Turin, Italy

## Abstract

**Background:**

Caprine herpesvirus 1 (CpHV-1) causes neonatal mortality and reproductive failure in goats. Despite its impact on herd reproductive performance, few studies have investigated the risk factors associated with CpHV-1 infection. The aim of this cross-sectional study was to identify potential herd- and host-level risk factors associated with CpHV-1 prevalence in a goat population with heterogeneous seropositivity for CpHV-1.

**Results:**

Blood samples and individual data from 4542 goats were collected from 255 herds in Piedmont, Italy. Enzyme-linked immunosorbent assay (ELISA) and serum neutralization tests were carried out to detect antibodies against CpHV-1. A mixed-effects model was applied to identify any statistical association between CpHV-1 seropositivity and a set of putative host-level and herd-level risk factors. A total of 630 samples tested were found positive by ELISA (prevalence = 13.9%; 95% confidence interval (CI) 12.9–14.9). Of the 255 tested herds, 85 were classified as positive for the presence of at least one gB-positive animal (herd prevalence 33.3%, 95% CI 27.5–39.2), with a within-herd prevalence between 0.7 and 100% (Q1 = 17.6%; median = 32.3%; Q3 = 50%) (Q = quartiles). The prevalence ratios showed a statistical association with the following risk factors: breeds other than Saanen, older age, larger herd size, meat and extensive herds, and co-existence of CAEV-infected animals.

**Conclusions:**

Results from this cross sectional study may help to elucidate the natural history of the infection and inform targeted strategies to control a disease with a potentially important impact on animal health and goat farming economy.

## Background

Caprine herpesvirus 1 (CpHV-1), an alphaherpesvirus antigenically closely related to bovine herpesvirus type 1 (BoHV-1), causes systemic disease and neonatal mortality in 1- or 2-week old kids [1] and reproductive failure in adult goats. A serological testing is often inconclusive due to the antigenic similarity of ruminant alphaherpesviruses related to BoHV-1 [2]. The percentage of nucleotide sequence identity for gB gene among different herpesviruses is indeed greater than 78% [3].

Even though BoHV-1 virus does not play a major role in small ruminants, both the natural infection in goat by Tolari *and coll.,* (1990) [4] and the susceptibility to experimental infection by Six *an coll.* (2001) [5] have been reported. Therefore ad hoc diagnostic strategies have been developed to discriminate the two viruses based on combination of ELISA tests [6–8].

CpHV-1 infects animals through the genital [9–11] or the respiratory mucosa [11] and establishes latent infection in sacral or trigeminal ganglia depending on the route of infection and the following spread through the body [12]. Although CpHV-1 infection is usually subclinical in adult goats, it can be responsible for different disorders including respiratory diseases, fever and leukopenia [10], vulvovaginitis [13, 14], balanoposthitis [15], and neonatal mortality. Abortions can be induced by the infection of pregnant goats at 3–4 months of gestation [16–19]. Severe disease may occur in neonatal kids characterized by pyrexia, conjunctivitis, oculonasal discharge, dyspnea, ulcerative and necrotic lesions throughout the enteric tract and high morbidity and mortality [20–22].

First isolated in the 1970s in California [22] and Switzerland [21] from young kids with severe generalised infection, CpHV-1 has been found worldwide since then in symptomatic animals from New Zealand [23], Australia [14, 15], Norway [24], and Greece [25]. More recently, CpHV-1 was isolated during an outbreak of infectious pustular vulvovaginitis in goats [13]. In Italy, it was first isolated from latently infected goats in 1996 [26]. Serological surveys indicate a worldwide distribution of CpHV-1 infection [14, 21, 22, 27, 28] and a widespread prevalence in Mediterranean countries where goats play an important economic role, with rates of more than 50% reported for Greece [25] and France [29], 36–43% for southern Italy [30, 31], and 21% for Spain [32].

In general, it is not clear if clinical disease outbreaks may go unreported or if the infection is lowly pathogenic: where prevalence is high unexplained abortions and reproductive disorder, such as infertility and return to estrus, might be associated with undiagnosed infections [33–35] and might cause substantial economic loss, especially in countries with many goat herds [20, 27, 32, 34, 36]. Despite its impact on herd reproductive performance, few studies have investigated the risk factors associated with CpHV-1 infection. To date, the use of natural mating, the herd size and the animal age have been reported as risk factor significantly positively associated with CpHV-1 prevalence [28, 33]. The aim of this cross-sectional study was to identify potential herd- and host-level risk factors associated with CpHV- prevalence in a goat population with heterogeneous seropositivity for CpHV-1.

## Results

A total of 630 out of 4542 caprine blood samples tested positive in the BoHV-1 gB blocking ELISA (prevalence 13.9%, 95% confidence interval (CI) 12.9–14.9); 5.5% of the positives (*n* = 35) tested positive also in the BoHV-1 gE blocking ELISA. On subsequent testing in the serum neutralization assay, all the 630 samples were classified as positive for CpHV-1 because of higher titers detected for this virus as compared with the titers for BoHV-1, by a factor of four dilutions. Of the 255 tested herds, 85 were classified as positive for the presence of at least one gB-positive animal (herd prevalence 33.3%, 95% CI 27.5–39.2), with a within-herd prevalence between 0.7 and 100% (Q1 = 17.6%; median = 32.3%; Q3 = 50%) (Q = quartiles).

Tables 1 and 2 show the results of the univariate analysis for host- and herd-risk factors, respectively. The prevalence ratios show a significant statistical association for all risk factors but sex. Both herd size and age showed a statistically linear dose-response trend with risk of infection. Table 3 presents the results of a multivariable mixed-effects Poisson regression model. Despite adjustment for confounding, all variables were still statistically associated with CpHV-1 infection except the presence of co-inhabitant cattle. This variable was therefore omitted in the last model.

**Table 1:** Association between CpHV-1 seropositivity and potential host-level risk factors as estimated by univariate analysis for caprine herpesvirus-1 infection in Piedmont (Italy). *N* = 4542 (PR = prevalence ratio)

Risk factor	Exposure level	No. of tested animals	No. of positive animals	Prevalence (%)	PR (95% CI)	*P*-values
Sex	Female	3762	581	15.4	Referent	
	Male	244	35	14.3	0.93 (0.66–1.3)	0.671
Breed	Saanen	629	9	1.4	Referent	
	Chamoisee	784	45	5.7	4.0 (2.0–8.2)	0.000
	Alpine	629	88	14.0	9.8 (4.9–19.4)	0.000
	All others	1901	466	24.5	17.1 (8.9–33.1)	0.000
Age^*^	1. (2–16 months)	914	47	5.1	Referent	
	2. (17–31 months)	913	80	8.8	1.7 (1.2–2.4)	0.004
	3. (32–56 months)	916	196	21.4	4.2 (3.0–5.7)	0.000
	4 (> 56 months)	917	265	28.9	5.6 (4.1–7.7)	0.000

^*^extension of the Wilcoxon rank-sum test for the age variable = 15.5, *P* value = 0.000

**Table 2:** Association between CpHV-1 seropositivity and potential herd-level risk factors as estimated by univariate analysis for caprine herpesvirus-1 infection in Piedmont (Italy). N = 4542 (PR = prevalence ratio)

Risk factor	Exposure level	No. tested animals	No. positive animals	Prevalence (%)	PR (95% CI)	*P*-values
Presence of CAEV-infected goats	No	1610	57	3.5	Referent	
	Yes	2132	428	20.1	5.7 (4.3–7.5)	0.000
Breeding type	Dairy	859	8	0.93	Referent	
	Meat/mixed	3581	613	17.1	18.4 (9.2–36.9)	0.000
Management type	Confined	1753	104	5.9	Referent	
	Extensive	2665	505	18.8	3.2 (2.6–3.9)	0.000
Herd size (no. of animals)^*^	1 (< 9 goats)	506	50	9.9	Referent	
	2 (9–21 goats)	777	99	12.7	1.3 (0.91–1.8)	0.143
	3 (> 21 goats)	3259	481	14.8	1.5 (1.1–2.0)	0.007
Presence of co-inhabitant cattle	No	2221	158	7.1	Referent	
	Yes	2321	472	20.3	2.9 (2.4–3.4)	0.000

^*^extension of the Wilcoxon rank-sum test for the herd size variable = 3.11; *P* value = 0.002

**Table 3:** The combined effect of risk factors according to the multivariable mixed-effects Poisson regression model for caprine herpesvirus-1 infection in Piedmont (Italy). *N* = 2879 (PR = prevalence ratio)

Risk factor	Exposure level	PR (95% CI)	*P*-values
Breed	Saanen	Referent	
	Chamoisee	2.9 (1.0–8.7)	0.053
	Alpine	4.2 (1.4–12.5)	0.009
	All others	4.4 (1.6–12.1)	0.005
Age	1 (2–16 months)	Referent	
	2 (17–31 months)	1.5 (1.0–2.2)	0.073
	3 (32–56 months)	2.9 (1.9–4.4)	0.000
	4 (> 56 months)	3.6 (2.3–5.5)	0.000
Breeding type	Dairy	Referent	
	Meat/mixed	11.5 (1.2–113.8)	0.037
Management type	Confined	1.0	
	Extensive	8.7 (2.8–27.2)	0.000
Herd size (n. of animals)	1 (< 9 goats)	Referent	
	2 (9–21 goats)	1.4 (0.7–3.0)	0.323
	3 (> 21 goats)	2.7 (1.4–5.3)	0.003
Presence of CAEV-infected goats	No	Referent	
	Yes	5.4 (2.3–12.8)	0.000

Likelihood-ratio test (mixed-effects model vs. fixed-effects model): *P*-value = 0.000

The goodness of fit of this model was better than that of a fixed effects model, as shown by the Akaike information criterion (AIC) values of 1683.8 and 1811.4, respectively, showing a significant herd random effect. In the final model, a total of 2879 records was considered because of missing data related to the presence of caprine arthritis and encephalitis virus (CAEV) infection in the herds (689 records). The latter variable was omitted in a further model including 3568 records; however, as compared with the previous one, the effect of all risk factors was still statistically significant (in particular meat/mixed goats showed a prevalence ratio of 12.2, 95% CI 1.9–78.9 and goats kept in extensive herds a prevalence ratio of 3.5, 95% CI 1.6–7.3).

## Discussion

This is the first study to seek associations between host- and herd-risk factors and CpHV-1 infection in Italian goats. The excess risk associated with several of these factors (breeds other than Saanen, older age, larger herd size, meat and extensive herds, and co-existence of CAEV-infected animals) was quantified by fitting a multivariable mixed-effects model that takes into account both the potential confounding effect of combined risk factors and the aggregation of animals in herds (herd random effect). While the risk factors examined here were similar to those known for bovine herpesvirus infections, CpHV-1 infection was also associated with two additional factors: meat/mixed breeding type and co-presence of CAEV-infected animals in the goat herds.

The association of the infection with herd size and age has been reported also in a recent study [33]. The effect of aging on the risk may be explained by prolonged exposure to the virus over time, with age acting as a surrogate measure of cumulative exposure. This holds true also for life-long seropositivity in BHV-1 infection [37–41]. As suggested by previous studies in cattle, the excess risk was also associated with herd size because larger herds are more likely to have cases since they have more animals at risk and more contacts with other herds through visits by animal handlers (e.g., veterinarians, farmers, inseminators) [37, 42–45]. The potential role of the number and frequency of new animal purchases could not be assessed because such data are, unfortunately, not recorded in the National Animal Registry system for sheep and goats. Finally, the smaller number of susceptible animals in small herds may limit the maintenance of infections over time [38].

The association with extensive running of a herd may, again, reflect a higher probability of transmission through contacts between susceptible and infected goats in situations where the farmer has less direct control over animal movement and behaviour. In contrast, the greater control typical of dairy goat herd management appears to confer dairy herds a protective effect, as seen even after having adjusted for breed. Generally, dairy farmers pay more attention to such health-related practices as selection of purchased animals and compliance with quarantine regulations and biosecurity standards. In meat herds the productive life of the animals is shorter and productivity is prioritized.

Moreover, the calculated risk for breed further confirms this observation. Most goats are reared for cheese production. Specifically, the Saanen breed is typically reared for the sale of fresh goat milk, which imposes stricter adherence to good biosecurity practices (pasture, reproduction, etc.) by dairy goat farms than farms where mixed breeds are reared. A greater or lesser susceptibility by breed cannot be excluded, however.

The strong association between the risk of herpesvirus and the presence of CAEV-infected animals highlights a potential for an epidemiological link between the two infections. Previous studies have found an association between bovine herpesvirus infection and other infectious diseases [38, 46] and showed that infectious diseases share common risk factors [45]. We cannot exclude that the presence of CAEV infection, which is frequently contracted in the first months of life without manifesting clinical symptoms [47, 48] may predispose infected animals to contracting other infectious diseases, and may also increase the probability of herpesvirus infection. Small ruminant lentivirus (SRLVs) does not infect lymphocytes, however, and immunodeficiency is not a significant feature in SRLV disease [49].

Finally, we think that the external validity of the results was not put at risk by the way the data has been collected: our cross-sectional dataset was not based merely on convenience sampling as the large majority of the sampled herds had been randomly recruited in the frame of the national brucellosis plan.

## Conclusions

What the study does suggest is that the type of herd most at risk for CpHV-1 infection is a large one, extensively reared, of the meat or mixed breeding type, and with CAEV co-infection and in which the eldest animals are more likely to be infected. Because most of these risk factors are common to several infectious diseases, veterinary services should promote effective prevention strategies that increase adherence to biosecurity standards and good herd management practices. Better knowledge of the risk factors for CpHV1 infection is needed. Our results may help to elucidate the natural history of the infection and inform targeted strategies to control a disease with a potentially important impact on animal health and goat farming economy.

## Methods

### Sample collection

This cross-sectional study was conducted in Piedmont, Italy’s second largest region in geographical area after Sicily, located in the northwest corner of the country where it borders with France and Switzerland. According to the latest National Animal Registry Office figures (as of 31/08/2013), the total regional goat population is 72,900 distributed across 7028 herds.

A total of 255 goat herds were sampled for this project from May 2008 to December 2010. These herds were comprised of 199 herds (78.0%) sampled as part of the national plan for brucellosis prevention (81 of these herds were also on the CAE eradication and monitoring program); 14 herds (5.5%) on the CAE programme but not part of the brucellosis programme; and 42 herds (16.5%) sampled for non-specified reasons. On these farms, a total of 4542 animals were blood sampled; individual animal data (sex, age and breed) was also collected. The herd size ranged from 1 to 143 animals (Q1–3; median = 8; Q3 = 21).

As an age requirement for being part of this study, only animals over two months of age were recruited. Information at the herd-level (breeding type, management type, herd size, etc.) was downloaded from the official database of the National Animal Registry system which is provided by the veterinary officers of the Ministry of Health through routine annual censuses.

### Serological testing for CpHV-1

The same approach as that described by Thiry et al. (2008) was applied to detect antibodies to CpHV-1 using an ELISA that has a specificity of 100% and a sensitivity of 93.5%.

### Elisa

BoHV-1 is serologically related to CpHV-1 [2]. CpHV-1 antibodies are known to cross-react with BoHV-1 antigens. A BoHV-1 ELISA was used following the approach suggested by Thiry et al. (2008). The serum samples were tested to detect antibodies to the highly conserved gB of alphaherpesviruses by means of a commercially available ELISA (Herdchek Anti-IBR gB, Idexx, Germany) following the manufacturer’s recommendations.

In order to rule out that the CpV-1 positivity might be due to BoHV-1, the samples testing positive for gB antibodies were also tested for gE antibodies using a BoHV-1 blocking ELISA (Herdchek Anti-IBR gE, Idexx). This combination with BoHV-1 gE blocking ELISA allows to distinguish between CpHV-1 and BoHV-1. This was demonstrated by Thiry et al. (2008), where CpHV-1-infected goats are determined seropositive by BoHV-1 gB blocking ELISA and seronegative by BoHV-1 gE blocking ELISA. We used a gB blocking ELISA different from that Thiry et al. used, but the overall performance of the diagnostic techniques are similar.

Samples showing an inhibition of < 45%, 45%–55%, and > 55% were classified as negative, doubtful, and positive, respectively. The serum samples classified as doubtful were subsequently analysed in a second round; those resulting doubtful on two tests were classified as CpHV-1 positive.

### Serum neutralization assay

Since in some cases goats may still be gE positive although infected by CpHV-1 [34], serum samples found positive in both ELISAs (*n* = 35) were subsequently tested using a cross-serum neutralization assay to detect antibodies to both the goat and the bovine viruses. Serum neutralization assays were carried out according to the procedure described in the Manual of Diagnostic Test and Vaccines for Terrestrial Animals [50]. Briefly, serum samples were heated to 56 °C for 30 min. Serial twofold dilutions of each serum sample were incubated at 37 °C for 2 h in the presence of 100 TCID50/cell of CpHV-1 Ba-1 strain [26] or 24 h in the presence of 100 TCID50/cell of BoHV-1 [50]. Each virus-serum dilution was then dispensed into 96 wells on microtiter plates containing Madin-Darby bovine kidney (MDBK) cells grown and incubated at 37 °C. The plates were read 3 days later. The titer of each serum is expressed as the highest dilution that neutralized the virus.

### Statistical analysis

Goat-level prevalence of CpHV-1 and within-herd prevalence were computed as the percentage of goat cases and relative 95% CI. We carried out a preliminary univariate analysis to identify any statistical association between CpHV-1 seropositivity (dependent variable) and potential risk factors: the respective prevalence in the exposed and the non-exposed animals was used to obtain prevalence ratios and relative 95% CI. When the 95% CI of the prevalence ratios did not include the null value (PR = 1), the findings were considered statistically significant.

Animal characteristics (sex, breed, and age) were used as host-level factors; herd-level factors were breeding type (dairy/meat/mixed), management type (extensive/confined), herd size, presence of CAEV-infected goats and of co-inhabitant cattle. As mentioned above, the herd-level data were obtained through a record linkage technique based on official herd identifiers to link our database with the National Animal Registry system. The databases and the data processes for statistical analysis were created with Stata Software 11.1 (Stata Corp, College Station, TX). Age and herd size were categorized into classes based on quartiles (first and second quartiles were conflated in a single category for herd size). The prevalence ratios were then estimated separately for each stratum using the lowest category as the reference. An extension of the Wilcoxon rank-sum test was used to evaluate trends in the prevalence ratios across strata of increasing age and herd size. For the “breed” variable, the less numerous breeds were grouped into a single class. Taking into account the cross-sectional study design and the clustering due to herds, we fit a mixed-effects Poisson regression model (*N* = 2879) with robust variance using the xtpoisson command to obtain adjusted prevalence ratios [51]. All variables that were statistically associated with CpHV-1 infection at the univariate analysis were then entered as independent variables, including “herd” as a random-effect variable. Finally, the AIC was applied to compare the mixed versus the fixed-effect models [52]**.** Due to the large proportion of missing values affecting the variable “presence of CAEV-infected goats”, a model omitting the variable was also fitted (*N* = 3568) in order to increase the number of total observations included in the analysis.

As all samples collected during the mentioned time window were considered for analysis, a prior calculation of the statistical power when comparing two independent proportions (i.e. in animals from the referent group and exposure level group) was carried out before the statistical analysis using WinPepi (version 11.65, http://www.brixtonhealth.com/pepi4windows.html): given a 95% significance level, thanks to the large available sample sizes (i.e. 2879 or 3568 goats respectively in the two final models), a 80% power could easily obtained to detect prevalence ratios between 1.5 and 2.

## Abbreviations

AIC: Akaike information criterion
BoHV-1: Bovine herpesvirus type 1
CAE: Caprine arthritis encephalitis
CAEV: Caprine arthritis and encephalitis virus
CI: Confidence interval
CpHV-1: Caprine herpesvirus 1
gB: Glycoprotein B
gE: Glycoprotein E
PR: Prevalence ratio
Q: Quartiles
SRLVs: Small ruminant lentivirus
